# Randomized phase III trial of regorafenib in metastatic colorectal cancer: analysis of the CORRECT Japanese and non-Japanese subpopulations

**DOI:** 10.1007/s10637-014-0154-x

**Published:** 2014-09-12

**Authors:** Takayuki Yoshino, Yoshito Komatsu, Yasuhide Yamada, Kentaro Yamazaki, Akihito Tsuji, Takashi Ura, Axel Grothey, Eric Van Cutsem, Andrea Wagner, Frank Cihon, Yoko Hamada, Atsushi Ohtsu

**Affiliations:** 1 Department of Gastroenterology and Gastrointestinal Oncology, National Cancer Center Hospital East, 6-5-1, Kashiwanoha, Kashiwa, 277-8577 Chiba Japan; 2 Department of Cancer Chemotherapy, Hokkaido University Hospital Cancer Center, Sapporo, Japan; 3 Gastrointestinal Oncology Division, National Cancer Center Hospital, Tokyo, Japan; 4 Gastrointestinal Oncology, Shizuoka Cancer Center, Nagaizumi-cho, Sunto-gun, Shizuoka, Japan; 5 Department of Medical Oncology, Kochi Health Sciences Center, Kochi, Japan; 6 Department of Clinical Oncology, Aichi Cancer Center Hospital, Chikusa-ku, Nagoya, Aichi Japan; 7 Department of Oncology, Mayo Clinic, Rochester, MN USA; 8 Digestive Oncology, University Hospitals and KU Leuven, Leuven, Belgium; 9 Global Clinical Development–Oncology, Bayer Pharma AG, Berlin, Germany; 10 Global Biostatistics, Bayer HealthCare Pharmaceuticals, Whippany, NJ USA; 11 Medical Affairs, Oncology & Hematology, Bayer Yakuhin Ltd, Osaka, Japan; 12 Exploratory Oncology Research & Clinical Trial Center, National Cancer Center Hospital East, Kashiwa, Chiba, Japan; 13 Present Address: Kobe City Hospital Organization, Kobe City Medical Center General Hospital, Kobe, Japan

**Keywords:** Regorafenib, Protein kinase inhibitors, Japanese, Colorectal cancer, Clinical trial, Phase III

## Abstract

*Background* In the international, phase III, randomized, double-blind CORRECT trial, regorafenib significantly prolonged overall survival (OS) versus placebo in patients with metastatic colorectal cancer (mCRC) that had progressed on all standard therapies. This *post hoc* analysis evaluated the efficacy and safety of regorafenib in Japanese and non-Japanese subpopulations in the CORRECT trial. *Methods* Patients were randomized 2 : 1 to regorafenib 160 mg once daily or placebo for weeks 1–3 of each 4-week cycle. The primary endpoint was OS. Outcomes were assessed using descriptive statistics. *Results* One hundred Japanese and 660 non-Japanese patients were randomized to regorafenib (*n* = 67 and *n* = 438) or placebo (*n* = 33 and *n* = 222). Regorafenib had a consistent OS benefit in the Japanese and non-Japanese subpopulations, with hazard ratios of 0.81 (95 % confidence interval [CI] 0.43–1.51) and 0.77 (95 % CI 0.62–0.94), respectively. Regorafenib-associated hand–foot skin reaction, hypertension, proteinuria, thrombocytopenia, and lipase elevations occurred more frequently in the Japanese subpopulation than in the non-Japanese subpopulation, but were generally manageable. *Conclusion* Regorafenib appears to have comparable efficacy in Japanese and non-Japanese subpopulations, with a manageable adverse-event profile, suggesting that this agent could potentially become a standard of care in patients with mCRC.

**Electronic supplementary material:**

The online version of this article (doi:10.1007/s10637-014-0154-x) contains supplementary material, which is available to authorized users.

## Introduction

Colorectal cancer (CRC) is the second most common cancer in Japan, with estimated age-standardized incidence and mortality rates of 32.2 and 11.9 per 100,000 population, respectively, in 2012 [1]. An estimated 1.4 million new cases of CRC occur worldwide each year, with almost 700,000 deaths attributed to the disease, resulting in CRC being one of the most important malignancies in the world [1].

More than 40 % of patients in Japan have locally advanced or metastatic CRC (mCRC) at diagnosis [2], for which standard therapy generally consists of chemotherapy based on a fluoropyrimidine plus either oxaliplatin or irinotecan, combined with anti-vascular endothelial growth factor (VEGF) therapy such as bevacizumab or ziv-aflibercept. Patients with *KRAS* wild-type tumors also receive cetuximab or panitumumab [3–5]. However, until recently, no further options were available for patients whose disease progressed on available standard therapies.

Regorafenib is an orally administered multikinase inhibitor that blocks the activity of a number of protein kinases associated with angiogenesis (VEGF receptors 1–3 and TIE2), oncogenesis (KIT, RET, RAF1, BRAF, and BRAF V600E) and the tumor microenvironment (platelet-derived growth factor receptor and fibroblast growth factor receptor) [6]. The international multicentre, phase III CORRECT (patients with metastatic COloRectal cancer treated with REgorafenib or plaCebo after failure of standard Therapy) trial compared regorafenib with placebo in patients with mCRC that had progressed on all available standard therapies or who were unable to tolerate standard therapies [7]. On the basis of the results of CORRECT, regorafenib was approved for the treatment of mCRC that has progressed on standard therapy by the US Food and Drug Administration in September 2012 [8], by the Japanese Ministry of Health, Labor, and Welfare in March 2013 [9], and by the European Medicines Agency in August 2013 [10].

CORRECT met its primary endpoint at a planned, formal interim analysis, with an overall survival (OS) hazard ratio (HR) for regorafenib versus placebo of 0.77 (95 % confidence interval [CI] 0.64–0.94; one-sided *p* = 0.0052) [7]. Progression-free survival (PFS) was also significantly greater in the regorafenib group than in the placebo group (HR 0.49, 95 % CI 0.42–0.58; one-sided *p* < 0.0001). Furthermore, regorafenib showed OS and PFS benefit in both *KRAS*-wild-type and *KRAS*-mutant tumor subgroups [7].

Previous trials of kinase inhibitors have shown differing adverse-event profiles in Japanese and non-Japanese patients. Subpopulation analyses of two international phase III studies comparing axitinib versus sorafenib and everolimus versus placebo in patients with metastatic renal cancer showed that Japanese patients had higher rates of several adverse events, including hand–foot skin reaction (HFSR), than the overall trial population [11, 12]. Therefore, it was felt to be important to investigate the safety and efficacy profile of regorafenib in Japanese and non-Japanese patients in the CORRECT trial.

This *post hoc* analysis was conducted to assess the efficacy, safety, population pharmacokinetics (PK), and quality-of-life (QoL) effects of regorafenib in the Japanese and non-Japanese subpopulations in the CORRECT trial. In addition, the potential relationship between patients’ baseline body size and the occurrence of regorafenib-associated adverse events was assessed.

## Materials and methods

CORRECT was a phase III, randomized, double-blind, placebo-controlled trial involving 114 centers in 16 countries. Eligibility criteria, treatment schedule, endpoints, and statistical analysis criteria have been reported in detail previously [7]. Included patients were aged at least 18 years and had mCRC that had progressed during or within 3 months after the last dose of all available standard therapies for mCRC. Patients were randomized 2 : 1 to receive regorafenib 160 mg or placebo once daily for weeks 1–3 of each 4-week cycle, in combination with best supportive care. The primary endpoint of the trial was OS (time from randomization to death from any cause), while secondary efficacy endpoints included PFS (time from randomization to first radiological or clinical progression or death from any cause), objective response rate (ORR), and disease control rate (DCR). Safety, health-related QoL, and PK were also assessed. The trial required 582 deaths for the final analysis. The first formal interim analysis, to assess futility, was conducted when approximately 30 % of the expected total number of deaths had occurred. The second interim analysis, at about 70 % of the total expected deaths, evaluated both efficacy and futility. Monitoring boundaries were based on prespecified O’Brien–Fleming-type stopping boundaries. The primary and secondary efficacy time-to-event endpoints for the overall population were analyzed using a one-sided log-rank test stratified by the same stratification factors as at randomization. The trial was registered at ClinicalTrials.gov, identifier NCT01103323.

*Post hoc* subpopulation analyses performed for Japanese and non-Japanese patients presented here were descriptive in nature, using Kaplan–Meier (KM) estimates and KM curves to analyze time-to-event parameters, such as OS and PFS. HR estimates with 95 % CIs were determined on the basis of an unstratified Cox regression model. For the subpopulation analyses, a HR (regorafenib over placebo) of less than 1 suggested a beneficial effect favoring regorafenib, whereas a HR of greater than 1 suggested an effect favoring placebo.

Health-related QoL was measured using the cancer-specific European Organisation for Research and Treatment of Cancer (EORTC) Quality of Life Questionnaire core-30 (QLQ-C30) [13], and the general-health EuroQol 5-dimension (EQ-5D) health utility index and visual analog scale (VAS) [14]. An analysis-of-covariance model was used to examine the time-adjusted area under the curve (AUC) of the EORTC QLQ-C30 global health status, EQ-5D health utility index, and EQ-5D VAS scores, with least-squares (LS) mean time-adjusted AUCs and 95 % CIs estimated for each treatment group and for the difference between treatment groups. QoL outcomes were also compared using descriptive statistics.

Blood samples for PK analysis were collected on day 15 of cycle 1 before administration of study drug, 2–4 h after administration, and 5–10 h after administration. The PK of regorafenib and its pharmacologically active metabolites, M2 (N-oxide metabolite) and M5 (N-oxide/N-desmethyl metabolite), were analyzed using a population PK model based on data collected during earlier studies of regorafenib. Regorafenib and metabolite PK data were pooled with concentration data from other studies of regorafenib monotherapy and analyzed using non-linear mixed-effects modeling. PK data were evaluated with historical data, without formal statistical analysis.

## Results

### Patient demographics and baseline disease characteristics

Results are based on the planned, formal interim analysis database, considered the final formal and definitive results, where the CORRECT trial met its primary endpoint showing that the regorafenib group had a statistically significant improvement in OS compared with placebo. A total of 933 non-Japanese patients were screened for inclusion at 95 centers in 15 countries between 30 April 2010 and 22 March 2011; of these, 660 patients were randomized to treatment with regorafenib (*n* = 438) or placebo (*n* = 222), while 656 received at least one dose of study treatment and were included in the safety population (regorafenib *n* = 435, placebo *n* = 221). The ethnicity of the non-Japanese subpopulation consisted of 593 white, 14 black or African American, 11 non-Japanese Asian, and 42 other or not reported. A total of 119 Japanese patients were screened for inclusion at 19 centers in Japan between 17 November 2010 and 10 February 2011; of these, 100 patients were randomized to treatment with regorafenib (*n* = 67) or placebo (*n* = 33), while 97 received at least one dose of study treatment and were included in the safety population (regorafenib *n* = 65, placebo *n* = 32). The flow of patients in the Japanese subpopulation through the trial is shown in Online Resource 1 (Fig. S1).

In general, the baseline characteristics of the Japanese subpopulation were similar to those of the non-Japanese subpopulation (Table 1), with the exception of lower median bodyweight, lower median body-mass index (BMI), and a slightly greater proportion of patients with an Eastern Cooperative Oncology Group (ECOG) performance status of 0 in the Japanese subpopulation than in the non-Japanese subpopulation.

**Table 1:** Baseline demographics and characteristics of the Japanese and non-Japanese patient subpopulations in the CORRECT trial

	Japanese subpopulation		Non-Japanese subpopulation	
	Regorafenib (*n* = 67)	Placebo (*n* = 33)	Regorafenib (*n* = 438)	Placebo (*n* = 222)
Median age (range), years	63 (44–79)	61 (34–75)	61 (22–82)	61 (25–85)
Sex, *n* (%)				
Men	47 (70)	16 (49)	264 (60)	137 (62)
Women	20 (30)	17 (52)	174 (40)	85 (38)
Median bodyweight (range), kg	62.6 (35.5–108.0)	58.2 (34.0–89.7)	73.5 (38.5–169.6)	77.0 (43.0–156.0)
Median body mass index (range), kg/m^2^	23.7 (15.4–34.3)	22.9 (13.9–29.9)	25.0 (14.4–47.3)	26.2 (17.2–50.4)
ECOG performance status, *n* (%)				
0	41 (61)	24 (73)	224 (51)	122 (55)
1	26 (39)	9 (27)	214 (49)	100 (45)
Primary site of disease, *n* (%)				
Colon	37 (55)	15 (46)	286 (65)	157 (71)
Rectum	28 (42)	18 (55)	123 (28)	51 (23)
Colon and rectum	2 (3)	0	28 (6)	14 (6)
Number of previous systemic anticancer therapies for metastatic disease, *n* (%)				
1–2	17 (25)	5 (15)	118 (27)	58 (26)
3	20 (30)	7 (21)	105 (24)	65 (29)
≥ 4	30 (45)	21 (64)	215 (49)	99 (45)
Time from diagnosis of metastatic disease to randomization, *n* (%)				
< 18 months	8 (12)	2 (6)	83 (19)	47 (21)
≥ 18 months	59 (88)	31 (94)	355 (81)	175 (79)
*KRAS* mutation, *n* (%)				
No	26 (39)	18 (55)	179 (41)	76 (34)
Yes	39 (58)	14 (42)	234 (53)	143 (64)
Unknown	2 (3)	1 (3)	25 (6)	3 (1)

Abbreviation: *ECOG*, Eastern Cooperative Oncology Group

### Treatment

Drug exposure in Japanese and non-Japanese subpopulations is shown in Table 2. The mean ± standard deviation (SD) overall duration of regorafenib treatment (including time off drug and dose interruptions) was 10.3 ± 7.9 weeks (median 7.3; range 0.3–28.9) in the Japanese subpopulation and 12.3 ± 10.0 weeks (median 7.3; range 0.3–47.0) in the non-Japanese subpopulation. The mean ± SD daily dose of regorafenib was 142.2 ± 20.2 mg (median 152.7; range 91.1–160.0) and 147.9 ± 18.3 mg (median 160.0; range 85.7–160.0), respectively.

**Table 2:** Drug exposure in Japanese and non-Japanese patients

	Japanese subpopulation		Non-Japanese subpopulation	
	Regorafenib (*n* = 65)	Placebo (*n* = 32)	Regorafenib (*n* = 435)	Placebo (*n* = 221)
Overall duration of treatment,^a^ weeks				
Mean ± SD	10.3 ± 7.9	7.3 ± 4.7	12.3 ± 10.0	7.9 ± 5.3
Median (range)	7.3 (0.3–28.9)	7.0 (0.6–22.9)	7.3 (0.3–47.0)	7.0 (0.6–38.6)
Actual daily dose, mg				
Mean ± SD	142.2 ± 20.2	158.3 ± 9.4	147.9 ± 18.3	159.4 ± 3.8
Median (range)	152.7 (91.1–160.0)	160.0 (107.0–160.0)	160.0 (85.7–160.0)	160.0 (121.6–160.0)
Dose intensity (%)				
Mean ± SD	69.3 ± 21.2	89.1 ± 17.7	80.4 ± 19.3	90.3 ± 16.1
Median (range)	71.4 (9.5–100.0)	98.8 (19.1–100.0)	83.3 (9.5–114.3)	100.0 (19.1–100.0)
Patients with drug-related adverse events leading to:				
Dose modification,^b^ *n* (%)	55 (84.6)	6 (18.8)	223 (51.3)	17 (7.7)
Permanent discontinuation, *n* (%)	9 (13.8)	0	32 (7.4)	3 (1.4)

^a^Includes time off drug and dose interruptions

^b^Includes dose interruptions and reductions

Abbreviation: *SD*, standard deviation

Mean ± SD regorafenib dose intensity, defined as the proportion of planned dose received, was 69.3 ± 21.2 % (median 71.4; range 9.5–100.0) in the Japanese subpopulation and 80.4 ± 19.3 % (median 83.3; range 9.5–114.3) in the non-Japanese population.

Regorafenib dose modifications due to drug-associated adverse events were more frequent in the Japanese than in the non-Japanese subpopulation, being reported in 84.6 and 51.3 % of patients, respectively. Rates of regorafenib discontinuation due to drug-associated adverse events were higher in the Japanese than in the non-Japanese subpopulation, but still low in both subpopulations (13.8 and 7.4 %, respectively).

Drug exposure in the Japanese and non-Japanese subpopulations receiving placebo (in terms of overall duration of treatment, actual daily dose, dose intensity, and discontinuation due to drug-related adverse events) was similar.

### Efficacy

**Fig. 1:**
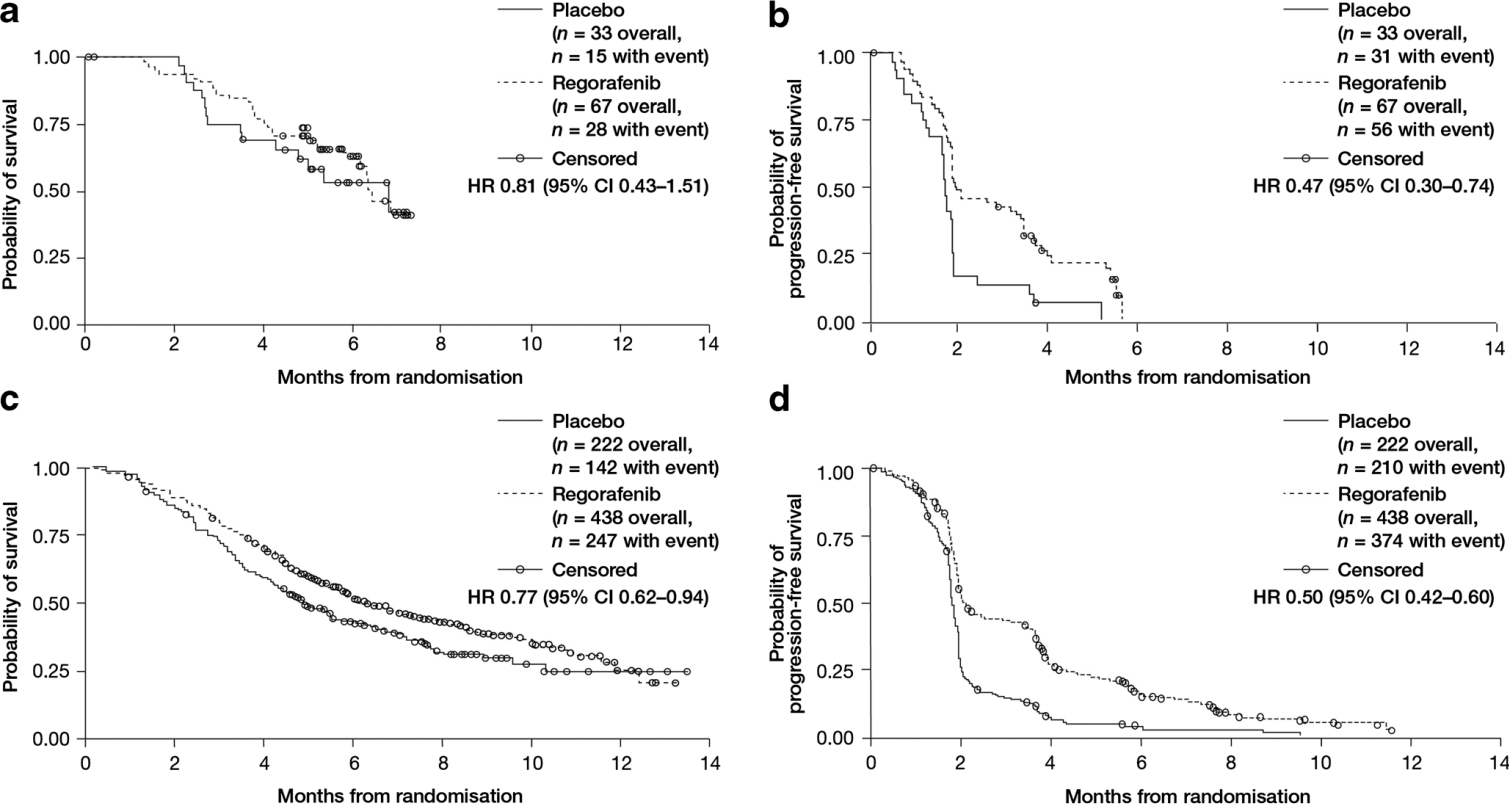
Kaplan–Meier curves showing overall survival and progression-free survival in Japanese (a and b, respectively) and non-Japanese patients (c and d, respectively)

The HR for OS in the regorafenib group vs placebo was 0.81 (95 % CI 0.43–1.51) in the Japanese subpopulation and 0.77 (95 % CI 0.62–0.94) in the non-Japanese subpopulation, with no apparent difference between subpopulations (Fig. 1a and c). The median OS in the regorafenib group was 6.6 months (range 0.2–7.5) in the Japanese subpopulation and 6.2 months (range 0.2–13.2) in the non-Japanese subpopulation, while median OS in the placebo group was 7.0 months (range 0.03–7.5) in the Japanese population and 4.9 months (range 0.4–13.6) in the non-Japanese subpopulation.

In both the Japanese and the non-Japanese subpopulations, PFS was greater in regorafenib-treated patients than in placebo recipients, with HRs of 0.47 (95 % CI 0.30–0.74) and 0.50 (95 % CI 0.42–0.60), respectively (Fig. 1b and d). The median PFS in regorafenib recipients was 1.9 months (range 0.03–5.7) in the Japanese subpopulation and 1.9 months (range 0.03–11.1) in the non-Japanese subpopulation, while median PFS in the placebo groups was 1.7 months (range 0.03–5.2) and 1.7 months (range 0.03–9.1) in the Japanese and non-Japanese subpopulations, respectively.

**Fig. 2:**
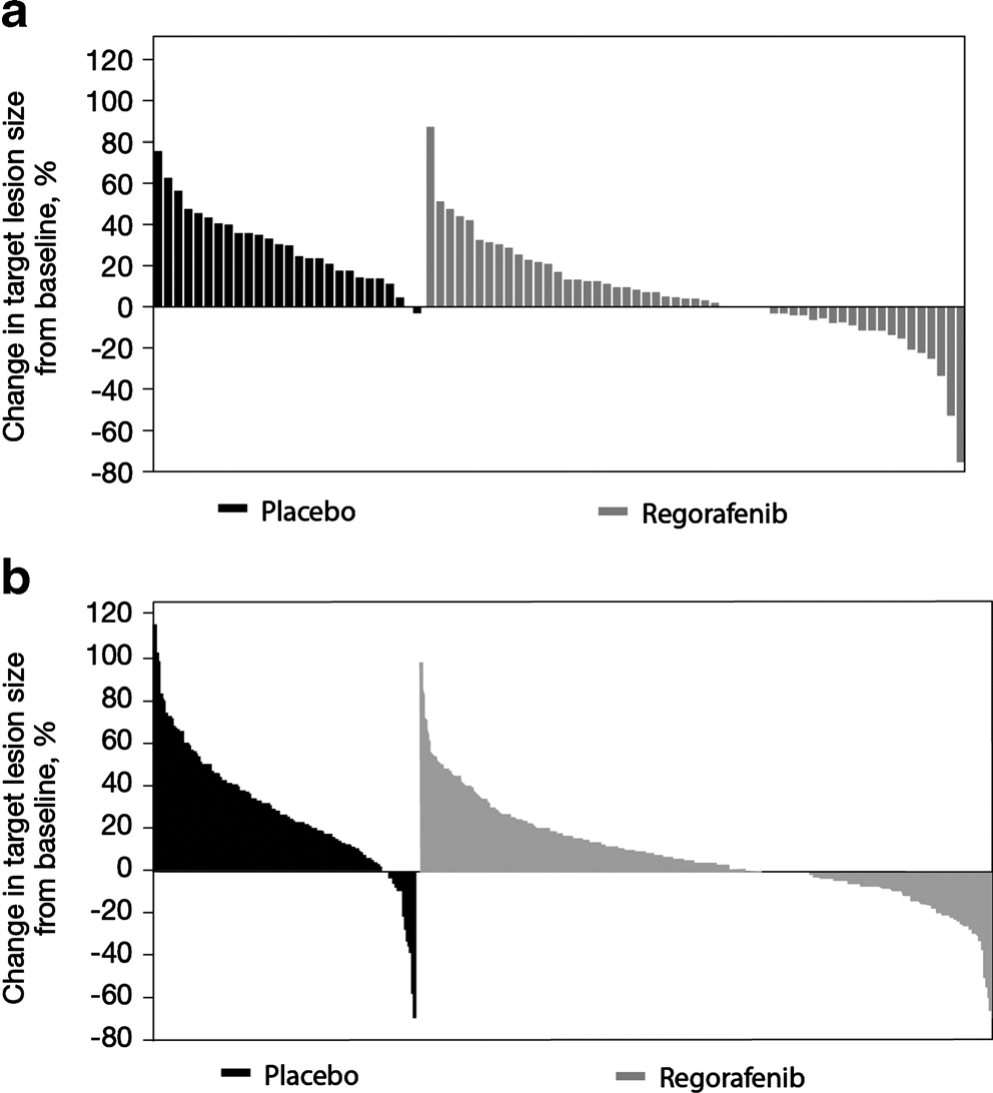
Maximum percentage change in target lesion size in (**a**) Japanese patients and (**b**) non-Japanese patients

In the regorafenib group, DCRs of 40 and 41 % were observed in the Japanese and non-Japanese subpopulations, respectively, compared with 15 % in both Japanese and non-Japanese subpopulations in the placebo group. Overall response was similar in the regorafenib and placebo groups, with ORRs of 2 and 1 % reported in the regorafenib-treated Japanese and non-Japanese subpopulations, respectively, consisting of partial responses in one Japanese patient and four non-Japanese patients. Among placebo recipients, no responses were reported in the Japanese subpopulation and one partial response (<1 %) was reported in the non-Japanese subpopulation. Reduction in tumor volume from baseline was observed in 33 % and 27 % of the regorafenib-treated Japanese and non-Japanese subpopulations, respectively. Changes in target lesion size in the Japanese and non-Japanese subpopulations are shown in Fig. 2.

### Safety

In the Japanese and non-Japanese subpopulations, treatment-related adverse events of any grade were observed in 99 and 92 % of regorafenib-treated patients, respectively, and in 59 and 61 % of placebo recipients, respectively (Table 3). Grade 3 or greater treatment-associated adverse events were reported in 80 and 51 % of regorafenib-treated patients, respectively, and 22 and 13 % of placebo recipients, respectively. No clear relationship was found between the incidence of regorafenib-associated adverse events and baseline BMI in the Japanese and non-Japanese subpopulation (Table 4).

**Table 3:** Drug-related adverse events reported in ≥10 % of patients receiving regorafenib or placebo in the Japanese and non-Japanese subpopulations

	Japanese subpopulation, *n* (%)				Non-Japanese subpopulation, *n* (%)			
	Regorafenib (*n* = 65)		Placebo (*n* = 32)		Regorafenib (*n* = 435)		Placebo (*n* = 221)	
Adverse event, *n* (%)	Any grade	Grade ≥3	Any grade	Grade ≥3	Any grade	Grade ≥3	Any grade	Grade ≥3
Any adverse event	64 (99)	52 (80)	19 (59)	7 (22)	401 (92)	223 (51)	135 (61)	28 (13)
Hand–foot skin reaction	52 (80)	18 (28)	1 (3)	0	181 (42)	65 (15)	18 (8)	1 (<1)
Hypertension	39 (60)	7 (11)	1 (3)	0	100 (23)	29 (7)	14 (6)	2 (<1)
Anorexia	28 (43)	6 (9)	8 (25)	3 (9)	124 (29)	10 (2)	31 (14)	4 (2)
Fatigue	28 (43)	5 (8)	8 (25)	3 (9)	209 (48)	43 (10)	63 (29)	10 (5)
Proteinuria	26 (40)	4 (6)	2 (6)	0	9 (2)	3 (<1)	2 (<1)	1 (<1)
Thrombocytopenia	25 (39)	4 (6)	2 (6)	0	38 (9)	10 (2)	3 (1)	1 (<1)
Rash/desquamation	23 (35)	2 (3)	4 (13)	0	107 (25)	27 (6)	6 (3)	0
Voice changes	21 (32)	0	0	0	126 (29)	1 (<1)	14 (6)	0
Fever	17 (26)	2 (3)	1 (3)	0	35 (8)	2 (<1)	6 (3)	0
Lipase elevation	16 (25)	9 (14)	0	0	8 (2)	7 (2)	1 (<1)	1 (<1)
Diarrhea	14 (22)	1 (2)	2 (6)	0	155 (36)	35 (8)	19 (9)	2 (<1)
Hypophosphatemia	13 (20)	9 (14)	0	0	12 (3)	10 (2)	1 (<1)	1 (<1)
Oral mucositis	13 (20)	1 (2)	0	0	123 (28)	14 (3)	9 (4)	0
Aspartate aminotransferase elevation	12 (19)	4 (6)	1 (3)	0	7 (2)	2 (<1)	4 (2)	2 (<1)
Nausea	11 (17)	1 (2)	4 (13)	0	61 (14)	1 (<1)	24 (11)	0
Epistaxis	10 (15)	0	1 (3)	0	26 (6)	0	4 (2)	0
Hyperbilirubinemia	10 (15)	1 (2)	2 (6)	1 (3)	35 (8)	9 (2)	2 (<1)	1 (<1)
Weight loss	10 (15)	0	1 (3)	0	59 (14)	0	5 (2)	0
Amylase elevation	9 (14)	2 (3)	0	0	5 (1)	2 (<1)	0	0
Constipation	9 (14)	0	0	0	33 (8)	0	12 (5)	0
Alanine aminotransferase elevation	8 (12)	3 (5)	1 (3)	0	4 (<1)	2 (<1)	1 (<1)	0
Taste alteration	7 (11)	0	0	0	28 (6)	0	5 (2)	0

**Table 4:** Relationship between regorafenib-associated adverse events and body mass index (BMI) in Japanese and non-Japanese patients

	Japanese population						Non-Japanese population					
	Regorafenib (*n* = 65)			Placebo (*n* = 32)			Regorafenib (*n* = 435)^a^			Placebo (*n* = 221)		
Adverse event, *n* (%)	BMI <25 kg/m^2^ (*n* = 44)	BMI 25 to <30 kg/m^2^ (*n* = 20)	BMI ≥30 kg/m^2^ (*n* = 1)	BMI <25 kg/m^2^ (*n* = 26)	BMI 25 to <30 kg/m^2^ (*n* = 6)	BMI ≥30 kg/m^2^ (*n* = 0)	BMI <25 kg/m^2^ (*n* = 210)	BMI 25 to <30 kg/m^2^ (*n* = 140)	BMI ≥30 kg/m^2^ (*n* = 73)	BMI <25 kg/m^2^ (*n* = 82)	BMI 25 to <30 kg/m^2^ (*n* = 86)	BMI ≥30 kg/m^2^ (*n* = 47)
Any adverse event	43 (98)	20 (100)	1 (100)	14 (54)	5 (83)	–	192 (91)	133 (95)	67 (92)	45 (55)	56 (65)	29 (62)
Hand–foot skin reaction	37 (84)	14 (70)	1 (100)	1 (4)	0	–	82 (39)	65 (46)	29 (40)	4 (5)	5 (6)	8 (17)
Hypertension	30 (68)	9 (45)	0	1 (4)	0	–	42 (20)	42 (30)	15 (21)	5 (6)	8 (9)	1 (2)
Anorexia	17 (39)	10 (50)	1 (100)	7 (27)	1 (17)	–	64 (30)	38 (27)	17 (23)	6 (7)	18 (21)	6 (13)
Fatigue	18 (41)	9 (45)	1 (100)	6 (23)	2 (33)	–	103 (49)	65 (46)	36 (49)	20 (24)	32 (37)	10 (21)
Proteinuria	19 (43)	6 (30)	1 (100)	2 (8)	0	–	1 (<1)	5 (4)	2 (3)	0	2 (2)	0
Thrombocytopenia	19 (43)	6 (30)	0	1 (4)	1 (17)	–	23 (11)	12 (9)	3 (4)	1 (1)	2 (2)	0
Rash/desquamation	14 (32)	9 (45)	0	2 (8)	2 (33)	–	59 (28)	36 (26)	12 (16)	1 (1)	4 (5)	1 (2)
Voice changes	16 (36)	5 (25)	0	0	0	–	51 (24)	53 (38)	18 (25)	5 (6)	5 (6)	3 (6)
Fever	9 (20)	8 (40)	0	1 (4)	0	–	22 (10)	8 (6)	5 (7)	3 (4)	3 (3)	0
Lipase elevation	10 (23)	6 (30)	0	0	0	–	3 (1)	4 (3)	1 (1)	0	1 (1)	0
Diarrhea	11 (25)	2 (10)	1 (100)	1 (4)	1 (17)	–	72 (34)	51 (36)	25 (34)	6 (7)	11 (13)	1 (2)
Hypophosphatemia	9 (20)	4 (20)	0	0	0	–	6 (3)	4 (3)	2 (3)	0	1 (1)	0
Oral mucositis	12 (27)	1 (5)	0	0	0	–	61 (29)	45 (32)	16 (22)	4 (5)	3 (3)	1 (2)
Aspartate aminotransferase elevation	7 (16)	5 (25)	0	0	1 (17)	–	6 (3)	1 (<1)	0	0	1 (1)	2 (4)
Nausea	8 (18)	3 (15)	0	3 (12)	1 (17)	–	38 (18)	15 (11)	6 (8)	5 (6)	9 (10)	8 (17)
Epistaxis	6 (14)	4 (20)	0	1 (4)	0	–	8 (4)	11 (8)	6 (8)	1 (1)	1 (1)	2 (4)
Hyperbilirubinemia	7 (16)	3 (15)	0	2 (8)	0	–	18 (9)	9 (6)	8 (11)	1 (1)	0	1 (2)
Weight loss	6 (14)	4 (20)	0	1 (4)	0	–	28 (13)	19 (14)	12 (16)	2 (2)	1 (1)	2 (4)
Amylase elevation	8 (18)	1 (5)	0	0	0	–	3 (1)	2 (1)	0	0	0	0
Constipation	6 (14)	3 (15)	0	0	0	–	9 (4)	16 (11)	6 (8)	3 (4)	6 (7)	3 (6)
Alanine aminotransferase elevation	5 (11)	3 (15)	0	0	1 (17)	–	3 (1)	0	1 (1)	0	0	1 (2)
Taste alteration	4 (9)	2 (10)	1 (100)	0	0	–	14 (7)	7 (5)	7 (10)	0	3 (3)	2 (4)

^a^Data on BMI were missing for 12 regorafenib-treated patients and six placebo recipients in the non-Japanese population

Regorafenib-associated adverse events of any grade occurring at a rate at least 20 % greater in the Japanese subpopulation than in the non-Japanese subpopulation were HFSR (80 vs 42 %), hypertension (60 vs 23 %), proteinuria (40 vs 2 %), thrombocytopenia (39 vs 9 %) and lipase elevations (25 vs 2 %). In contrast, the incidence of diarrhea was lower in the Japanese subpopulation than in the non-Japanese subpopulation (any grade: 22 vs 36 %; grade ≥3: 2 vs 8 %).

Serious drug-associated adverse events were reported in 9 and 12 % of the regorafenib-treated Japanese and non-Japanese subpopulations, respectively, compared with 9 and 3 % of the Japanese and non-Japanese subpopulation receiving placebo, respectively. The most frequent regorafenib-associated serious adverse event in the Japanese subpopulation was anorexia, reported in three patients (5 %).

Regorafenib-associated liver function adverse events were reported more frequently in the Japanese subpopulation than in the non-Japanese subpopulation, although the incidence of grade 3–4 liver events was low in both subpopulations. Alanine aminotransferase (ALT) elevations were reported in 12 % and fewer than 1 % of the regorafenib-treated Japanese and non-Japanese subpopulations, respectively, aspartate aminotransferase (AST) elevations in 19 and 2 %, respectively, and bilirubin elevations in 15 and 8 %, respectively. One case of lethal liver dysfunction related to regorafenib was observed in the Japanese subpopulation. The patient was a 62-year-old man with liver metastases and an ECOG performance status of 0 at trial entry. Forty-three days after starting regorafenib treatment, the patient presented with grade 3 jaundice and grade 2 liver dysfunction, and regorafenib treatment was permanently discontinued the following day. Laboratory test results showed elevated alkaline phosphatase (634 U/L; local laboratory range not provided), total bilirubin (7.7 × upper limit of normal [ULN] [9.2 mg/dL]), AST (30.8 × ULN [1261 U/L]) and ALT (31 × ULN [1393 U/L]). The patient’s platelet count was also depressed (91 × 10^3^/mm^3^; range not provided). The patient’s hepatitis status was confirmed as negative. The severity of liver dysfunction increased to grade 5 at 43 days after treatment discontinuation.

### Population pharmacokinetics

**Fig. 3:**
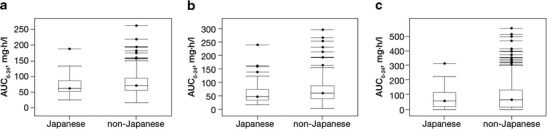
Distribution of area under the concentration–time curve over a 24-h dosing interval (AUC_0–24_) of (**a**) regorafenib, (**b**) M2 and (**c**) M5 in Japanese and non-Japanese patients. Boxes represent the median and interquartile range of AUC_0–24_ of regorafenib, M2 and M5; whiskers represent the range between the lowest and highest values within 1.5 times the first and third quartiles. Dots represent outlying values greater than 1.5 times the third quartile

Data from 381 patients (Japanese *n* = 41; non-Japanese *n* = 340) were included in the population PK analysis. The population PK analysis was based on 3,696 data points from samples collected before administration, 2–4 h after administration, and 5–10 h after administration of regorafenib on day 15 of cycle 1. The analysis showed a similar distribution of calculated AUC over a 24-h dosing interval and AUC from the start of treatment until 24 h after the last dose for regorafenib and its M2 and M5 metabolites in the Japanese and non-Japanese subpopulations (Fig. 3 and Online Resource 1 [Table S1]).

### Quality of life

LS mean time-adjusted AUCs of EORTC QLQ-C30 global health status, EQ-5D health utility index, and EQ-5D VAS scores did not differ between regorafenib and placebo groups in either the Japanese or the non-Japanese subpopulation. QoL scores appeared to be similar in the regorafenib and placebo groups at baseline and at the end of treatment in both the Japanese and non-Japanese subpopulations (Table 5).

**Table 5:** Time-adjusted area under the curve (AUC) and change from baseline in health-related quality-of-life scores in Japanese and non-Japanese patients

Outcome	Japanese population				Non-Japanese population			
	*n*	Regorafenib (*n* = 67)	*n*	Placebo (*n* = 33)	*n*	Regorafenib (*n* = 438)	*n*	Placebo (*n* = 222)
EORTC QLQ-C30 global health status								
Baseline, median (range)	65	75.0 (0.0, 100.0)	32	79.2 (16.7, 100.0)	411	66.7 (0.0, 100.0)	208	66.7 (0.0, 100.0)
End of treatment, median (range)	52	50.0 (0.0, 100.0)	30	50.0 (0.0, 91.7)	222	50.0 (0.0, 100.0)	114	50.0 (0.0, 100.0)
Time-adjusted AUC, LSM (95 % CI)		58.0 (53.1, 62.9)		57.7 (51.4, 63.9)		56.3 (52.0, 60.5)		57.7 (53.4, 62.0)
Difference from placebo		0.3 (−5.5, 6.2)		–		−1.4 (−3.5, 0.6)		–
EQ-5D health utility index								
Baseline, median (range)	64	0.8 (−0.1, 1.0)	31	0.9 (0.2, 1.0)	409	0.7 (−0.3, 1.0)	208	0.8 (−0.2, 1.0)
End of treatment, median (range)	51	0.7 (−0.4, 1.0)	29	0.7 (−0.4, 1.0)	221	0.7 (−0.6, 1.0)	114	0.7 (−0.3, 1.0)
Time-adjusted AUC, LSM (95 % CI)		0.7 (0.7, 0.8)		0.7 (0.6, 0.8)		0.6 (0.6, 0.7)		0.6 (0.6, 0.7)
Difference from placebo		0.0 (−0.0, 0.1)		−		−0.0 (−0.0, 0.0)		−
EQ-5D VAS								
Baseline, median (range)	65	75.0 (10.0, 100.0)	32	80.0 (30.0, 95.0)	406	69.0 (3.0, 100.0)	203	70.0 (4.0, 100.0)
End of treatment, median (range)	51	55.0 (4.0, 100.0)	30	60.0 (0.0, 99.0)	221	60.0 (0.0, 99.0)	113	60.0 (5.0, 100.0)
Time-adjusted AUC, LSM (95 % CI)		63.3 (59.2, 67.4)		65.7 (60.5, 70.9)		58.5 (54.5, 62.5)		59.5 (55.4, 63.7)
Difference from placebo		−2.4 (−7.3, 2.5)		−		−1.1 (−3.0, 0.9)		−

Abbreviations: *CI*, confidence interval; *EORTC QLQ*-*C30*, European Organisation for Research and Treatment of Cancer Quality of Life Questionnaire core-30; *EQ*-*5D*, EuroQol 5-Dimension; *LSM*, least-squares mean; *VAS*, visual analogue scale

## Discussion

CORRECT is the first randomized, controlled, phase III trial with a substantial number of Japanese patients to assess a new therapeutic agent in mCRC, with previous international phase III studies in this field including only a small number of Japanese patients or none at all. In this analysis, the primary endpoint of OS appeared similar in the Japanese and non-Japanese subpopulations, with HRs (regorafenib over placebo) of 0.81 and 0.77, respectively. Regorafenib was also associated with a greater improvement in PFS and higher DCRs than placebo, with consistent benefits in both the Japanese and the non-Japanese subpopulations.

The adverse-event profile of regorafenib in the Japanese subpopulation differed from that observed in the non-Japanese subpopulation, with a higher incidence of HFSR, hypertension, proteinuria, thrombocytopenia, and lipase elevations, and a lower incidence of diarrhea seen in the former group. The incidence of grade 3 or greater treatment-associated adverse events was higher in the Japanese than in the non-Japanese subpopulation, although serious regorafenib-associated adverse events were reported at similar rates in both subpopulations (9 vs 12 %, respectively) and no unexpected safety signals were observed in either. A higher incidence of HFSR in Japanese versus non-Japanese patients has been noted in previous clinical trials of other kinase inhibitors [11], as has a lower incidence of treatment-related diarrhea in Japanese versus non-Japanese patients with mCRC receiving fluoropyrimidine alone or in combination with irinotecan [15, 16]. Investigation of the relationships between the incidence of regorafenib-associated adverse events and BMI or body surface area in the Japanese and non-Japanese subpopulations found no clear trends (body surface area data not shown). Previous anti-epidermal growth factor receptor or capecitabine treatment also showed no relationship with regorafenib-associated HFSR in both the Japanese and the non-Japanese subpopulations (data not shown). Further investigation is needed to clarify the reasons for the higher incidence of HFSR, as well as other differences in adverse-event profile, in the Japanese versus the non-Japanese subpopulation.

One Japanese patient developed fatal liver dysfunction associated with regorafenib treatment, with onset 43 days after starting regorafenib, and death occurring 86 days after initiation of regorafenib. Overall, elevation of ALT, AST and bilirubin levels appeared to occur more frequently in the Japanese subpopulation than in the non-Japanese subpopulation, although rates of grade 3 or greater elevations appeared generally similar between subpopulations. Frequent and careful monitoring of liver function is advisable, especially during the first two cycles of treatment, because this period is when liver-function abnormalities occurred most frequently in our study.

Corresponding to the higher rate of adverse events observed in the Japanese subpopulation than in the non-Japanese subpopulation, dose modifications due to adverse events were more frequent in the regorafenib-treated Japanese subpopulation than in the non-Japanese subpopulation (84.6 vs 51.3 %, respectively), leading to the lower mean dose intensity in the former than in the latter group (69.3 vs. 80.4 %, respectively).

Rates of treatment discontinuation because of regorafenib-associated adverse events were higher in the Japanese subpopulation than in the non-Japanese subpopulation (13.8 vs 7.4 %, respectively). This difference might be due to interpatient PK variability; however, the regorafenib dose and schedule (160 mg once daily for the first 3 weeks of each 4-week cycle) that was selected as the recommended dose for further study in phase III trials, including CORRECT [7], was based on findings in a phase I trial in non-Japanese patients [17, 18], which was confirmed by a Japanese phase I trial [19]. In addition, population PK analysis in the CORRECT trial showed a similar distribution of regorafenib AUC over a 24-h dosing period in the Japanese and non-Japanese subpopulations. Further investigation is warranted to confirm the reason for the differences in adverse-event profiles and treatment tolerability between Japanese and non-Japanese populations.

Time-adjusted AUCs of the scores on the health-related QoL instruments appeared to be generally similar in both subpopulations, and did not differ significantly between regorafenib and placebo recipients in either subpopulation. Furthermore, differences between regorafenib and placebo were below the minimum difference in scores considered to be clinically meaningful [20, 21]. These findings indicate that, despite a higher rate of adverse events compared with placebo, regorafenib was not associated with clinically meaningful impairment of QoL in either the Japanese or the non-Japanese subpopulation.

It should be recognized that this subpopulation analysis has some limitations. Firstly, this analysis was not prespecified, although geographic differences were considered in the trial design, and geographical region was a stratification factor during randomization. Secondly, the Japanese subpopulation represents only a small proportion (*n* = 100) of the overall patient population (*n* = 760 total randomized patients) included in the CORRECT trial; as a result, subpopulation analyses had insufficient statistical power for any inferential analyses, which needs to be taken into consideration when interpreting the results. The trial was not designed to test for statistically significant differences between treatment effects (i.e., regorafenib vs placebo) in the Japanese and non-Japanese subpopulations. However, this subpopulation analysis may provide a clearer picture of the efficacy and safety of regorafenib in Japanese patients than comparisons with historical controls derived from other trials conducted in separate populations of Japanese and non-Japanese patients. A phase III trial entitled ‘Asian subjects with mCRC treated with regorafenib or placebo after failure of standard therapy (CONCUR)’ (ClinicalTrials.gov identifier: NCT01584830) is providing more robust efficacy and safety data for regorafenib in Asian patients.

In conclusion, regorafenib appears to be as effective in the Japanese subpopulation as it is in the non-Japanese subpopulation. The adverse-event profile was generally as expected in Japanese patients, although there were some differences in the occurrence of certain adverse events between the Japanese and non-Japanese subpopulations. Adverse events were generally manageable with dose modifications, and durations of treatment were similar in both subpopulations. On the basis of these results, regorafenib has the potential to become a standard of care for Japanese as well as non-Japanese patients with mCRC.

## Electronic supplementary material

Below is the link to the electronic supplementary material.

ESM 1 (DOCX 22 kb)

ESM 2 (DOCX 51 kb)
