# Supplementary material for: Randomized phase III trial of regorafenib in metastatic colorectal cancer: analysis of the CORRECT Japanese and non-Japanese subpopulations
Source: Invest New Drugs. 2014 Sep 12;33(3):740–50. doi: 10.1007/s10637-014-0154-x (PMC4434855; doi:10.1007/s10637-014-0154-x)
Supplement: Supplementary file 1 — (DOCX 22 kb) [file 10637_2014_154_MOESM1_ESM.docx]

**Table S1** Area under the concentration–time curve of regorafenib and its active metabolites in Japanese and non-Japanese patients

|  | **Japanese population (*n=*41)** | | **Non-Japanese population (*n=*340)** | |
| --- | --- | --- | --- | --- |
|  | **AUC_0–24_ (mg·h/l)** | **AUC_t1–t2_ (mg·h/l)** | **AUC_0–24_ (mg·h/l)** | **AUC_t1–t2_ (mg·h/l)** |
| **Regorafenib** | | | | |
| Mean ± SD | 72.7 ± 32.4 | 3545.3 ± 2331.0 | 78.9 ± 31.5 | 4680.3 ± 4111.5 |
| Median (range) | 62.8 (27.4–189.0) | 2790.0 (772.0–9340.0) | 72.7 (19.2–264.0) | 3360.0 (528.0–28800.0) |
| **M2** | | | | |
| Mean ± SD | 63.0 ± 48.1 | 2525.5 ± 2139.7 | 69.1 ± 42.4 | 3684.7 ± 3868.0 |
| Median (range) | 46.3 (16.6–237.0) | 1670.0 (467.0–9590.0) | 62.4 (3.5–295.0) | 2475.0 (163.0–36300.0) |
| **M5** | | | | |
| Mean ± SD | 81.3 ± 74.3 | 2745.9 ± 4492.5 | 97.6 ± 98.3 | 9115.8 ± 34837.6 |
| Median (range) | 60.5 (4.1–315.0) | 1270.0 (177.0–23200.0) | 71.3 (1.9–555.0) | 1920.0 (37.1–495000.0) |

Abbreviations: AUC_0–24_, area under the concentration–time curve over a 24-hour dosing interval

AUC_t1–t2_, area under the concentration–time curve from the start of treatment until 24 hours after the last dose

SD, standard deviation
