# Supplementary material for: Randomized phase III trial of regorafenib in metastatic colorectal cancer: analysis of the CORRECT Japanese and non-Japanese subpopulations
Source: Invest New Drugs. 2014 Sep 12;33(3):740–50. doi: 10.1007/s10637-014-0154-x (PMC4434855; doi:10.1007/s10637-014-0154-x)
Supplement: Supplementary file 2 — (DOCX 51 kb) [file 10637_2014_154_MOESM2_ESM.docx]

**Figure S1** Flow of Japanese patients through the CORRECT study

Assessed for eligibility
(*n*=119)

Randomised (*n*=100)

Excluded (*n*=19)

- Did not meet inclusion criteria (*n*=18)
- Adverse event (*n*=1)
- Allocated to regorafenib (*n*=67)
- Received regorafenib (*n*=65)
- Allocated to placebo (*n*=33)
- Received placebo (*n*=32)

Discontinued treatment (*n*=31)

- Adverse event associated
  with clinical progression
  (*n*=2)
- Radiological disease progression (*n*=29)

Discontinued treatment (*n*=56)

- Death (*n*=1)
- Patient withdrawal (*n*=3)
- Adverse event (*n*=9)
- Adverse event associated with clinical progression (*n*=5)
- Radiological disease progression (*n*=37)
- Clinical disease progression
  (*n*=1)

Included in efficacy analysis (intention-to-treat population; *n*=67)

Included in safety analysis (*n*=65)

Included in efficacy analysis (intention-to-treat population; *n*=33)

Included in safety analysis (*n*=32)
